# Sex differences in compulsive sexual behavior disorder

**DOI:** 10.3389/fnbeh.2026.1821534

**Published:** 2026-05-26

**Authors:** Aviv M. Weinstein

**Affiliations:** Department of Psychology, Ariel University, Ariel, Israel

**Keywords:** compulsive sexual behavior, hypersexuality, sex differences, sexual arousal, sex addiction

## Abstract

**Background:**

Compulsive sexual behavior is a failure to control intense, repetitive sexual impulses or urges, resulting in sexual behavior that causes marked distress or impairment. Studies on compulsive sexual behavior report a higher prevalence of CSBD among men. There are sex differences in the symptoms and behaviors associated with CSB, like sexual arousal and motivation.

**Methods:**

A systematic search in PubMed/Medline and Scopus databases between January 2000 and January 2026, using search terms related to compulsive sexual behavior.

**Results:**

While CSBD is more common in men, neuroticism and stress vulnerability contribute importantly to CSBD symptoms in women. Human sexuality is associated with neurotransmitters, hormones, and genes, in particular genes responsible for dopamine, which is associated with novelty seeking, impulsivity, and may influence sexual activity. Neuroimaging studies outlined sex differences in neural responses to sexual stimuli in men in fMRI. Men with CSBD showed an activation of reward areas in the brain in response to sexually explicit stimuli. Their higher sensitivity to the rewarding value of sexual cues may contribute to their increased risk of addictive or compulsive sexual behaviors. Studies on CSBD indicate that impulsivity and psychiatric comorbidity are common across both genders. Finally, childhood trauma and abuse are often reported in individuals seeking treatment for sex addiction, particularly among women.

**Implications:**

Little is known about sex differences in CSBD. Although childhood adversities may contribute to the development of CSB in both women and men, further research is needed to determine whether the groups respond differently.

## 1 Introduction

### 1.1 Diagnosis, behavioral manifestations, and related traits

Excessive sexuality had been recognized as a distinctive pathology since the beginning of the first millennium. Over time, a variety of constructs have emerged to describe compulsive sexual behavior, including *excessive sexuality, hyper-sexuality, sexual addiction*, and *out-of-control sexual behavior* (Sassover and Weinstein, 2022). Hypersexuality was introduced in the DSM-5, but it was not accepted due to a lack of sufficient clinical evidence, research, and concerns about a potential misuse in forensic settings. Sex addiction was highly controversial and raised serious criticism (Kor et al., 2013; Kraus et al., 2016a; Sassover and Weinstein, 2022). The 10th Edition of the International Classification of Diseases (ICD-10) used the term “excessive sexual drive” (World Health Organization, 2016). In 2019, Compulsive Sexual Behavior Disorder (CBSD) was officially included in the 11th Revision of the ICD as an impulse-control disorder (ICD-11; World Health Organization, 2019). CSBD was defined as “a persistent failure to control intense, repetitive sexual impulses or urges, resulting in repetitive sexual behavior that causes marked distress or impairment in personal, familial, social, educational, or occupational functioning for a period of at least 6 months” (World Health Organization, 2019). Compulsive Sexual Behavior (CSB) includes frequent sexual activities, problematic pornographic use, cybersex, and uncontrolled sexual thoughts and behaviors (Walton et al., 2017a). This behavior is also related to impulsivity and personality traits, including narcissism. There is general agreement that sex and gender are two distinct concepts: sex refers primarily to biological factors, whereas gender closely aligns with social norms and roles (Kaufman et al., 2023).

Sex and gender are important factors in brain research, in particular on sexual behavior. Sexual behavior is affected by hormones and neurotransmitters. Sex differences affect brain activity, in particular during sexual arousal. Visual sexual stimuli activate the cortico-limbic circuit, resulting in changes in emotion, reward, and memory, may have different effects on men and women. Furthermore, sex differences in decision-making and impulsivity may affect behavioral patterns of reward, cognitive control, and risk-taking behavior. This is particularly important for the understanding of compulsive sexual behavior, which is characterized as an impulse control disorder. Due to the importance of sex differences in sexual arousal and sexual behavior, this review sets out to investigate the neurobiological basis of sex differences in sexual arousal and compulsive sexual behavior. In this review, we will evaluate biological sex differences, including neuroendocrine, structural, and functional differences and their behavioral expressions. Table 1 describes the evolution of the constructs of compulsive sexual behavior and its clinical diagnosis.

**TABLE 1:** The historical evolution of compulsive sexual behavior.

Time	Description	Criticism
19th century origins	Early discussions introduced terms like “nymphomania” for women), and “satyriasis” for men, perceived as brain-related physical disorders.	These terms were criticized for their outdated, biased, and stigmatized nature, lacking of scientific and clinical accuracy, leading to their eventual abandonment.
Mid-20th century	The notion of “sexual addiction” emerged in the 1970s and 1980s, popularized by 12-step programs and Patrick Carnes’s book “The sexual addiction” by Carnes (1983).	The concept faced substantial criticism due to insufficient scientific evidence to recognize it as a clinical disorder. Critics argued that it pathologized normal sexual behavior, obscured underlying issues like anxiety, depression, and lacked consistent diagnostic criteria.
DSM-III-R (American Psychiatric Association, 1987)	The idea of “sexual addiction” was briefly considered.	It was not formally accepted, criticized for lacking scientific evidence to be classified as a clinical disorder.
1990s	Coleman (1990) introduced “Compulsive Sexual Behavior” as a response to anxiety, where sexual acts provided temporary relief, overlapping with OCD.	Coleman’s conceptualization was criticized for its ambiguous boundaries with obsessive-compulsive disorders (OCD) and for labeling normal sexual activity as pathological, lacking validated diagnostic criteria to distinguish it from conditions like bipolar disorder or normal sexual desire.
DSM-5 (American Psychiatric Association, 2013)	“Hypersexual Disorder” was proposed by Kafka (2010) to replace “sexual addiction.”	It was excluded due to insufficient clinical evidence, lack of research consensus, and concerns about potential misuse in forensic contexts.
ICD-10 (World Health Organization, 2016)	The 10th edition of the International Classification of Diseases (ICD-10) used “excessive sexual drive” within “other sexual dysfunctions, not caused by organic disorder or disease,” including nymphomania (female) and satyriasis (male), marking a high-desire sexual disorder.	The use of “nymphomania” and “satyriasis” was viewed as archaic and sexist by many clinicians. Classifying sexual urges as “excessive” was deemed subjective, often rooted in cultural norms rather than scientific dysfunction evidence, stigmatizing individuals. There was diagnostic overlap with other sexual preference disorders or high libido.
ICD-11 (World Health Organization, 2019)	The World Health Organization (WHO) formally recognized Compulsive Sexual Behavior Disorder (CSBD), classifying it as an impulse control disorder, not as an addictive disorder. Definition: “A persistent pattern of failure to control intense, repetitive sexual impulses or urges, causing significant distress or impairment in personal, family, social, or occupational functioning.” Symptoms must persist for an extended period, typically 6 months or more. The diagnosis is not made if behavior is better explained by another mental disorder (e.g., bipolar disorder).	Ongoing debates persist about its classification, with discussions on whether it should be categorized as a behavioral addiction or under obsessive-compulsive disorders.

## 2 Methodology

### 2.1 Search strategy

**FIGURE 1:**
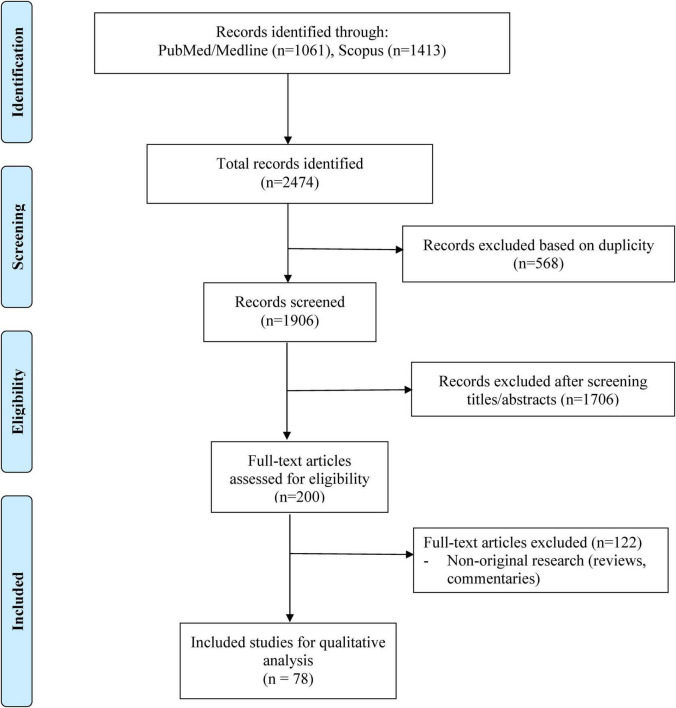
PRISMA flowchart of study selection and exclusion.

A systematic search in PubMed/Medline and Scopus databases was performed to identify peer reviewed studied published between January 2000 and January 2026, using the keywords variations. Search terms included combinations of “compulsive sexual behavior,” “hypersexuality,” “sexual arousal,” and “Sex differences.” English-language articles involving adults and young adults (ages 18+) with a focus on cross-sectional, longitudinal, and cohort studies were initially screened, followed by a full-text review to confirm relevance and adherence to inclusion criteria. In total, 78 studies and reviews were included: 20 focusing on epidemiology, 24 on neurotransmitters and hormones, 18 on brain imaging, 2 on impulsivity and personality, 11 on sexual abuse and sexual narcissism, and 3 on Pornography and cybersex. PRISMA flowchart outlining the selection process described in Figure 1.

## 3 Epidemiology- the behavioral expression of CSBD

CSB affects men more frequently than women, a difference that may be influenced by cultural factors. Cross-sectional studies based on convenience samples reported a higher prevalence of CSBD among men (see Kürbitz and Briken, 2021, for a review). In most studies, the percentage of individuals with CSBD was approximately 20%, with a male-to-female ratio of 3:1 or 4:1 (Engel et al., 2019; Walton et al., 2017b). Similar trends were observed in online populations of students (Vaillancourt-Morel et al., 2017; Kingston et al., 2018; Castro-Calvo et al., 2020; Dickenson et al., 2018), clients of a fitness center (Müller et al., 2015), and users of online dating applications (Shimoni et al., 2018; Levi et al., 2020). Several large population-based studies on CSBD confirmed a majority of men with CSBD. In a large study of 18,034 participants (34% females), LGBTQ males had the highest CSBD scores, and heterosexual females had the lowest (Bõthe et al., 2018). A large study of 16,823 Hungarian adults, reported that younger, homosexual males who were single, without full-time employment, and living in an urban city were at higher risk for CSB (Slavin et al., 2020a). Data from the International Sex Survey of 82,243 individuals across 42 countries revealed that approximately 5% were at high risk for CSBD. Men scored highest on CSBD, followed by gender-diverse individuals and women (Bõthe et al., 2023).

Population-based studies on problematic pornography use also confirmed a majority of men. A survey of 14,006 individuals who had viewed pornography in the past year, reported 30% women (Bõthe et al., 2020). Finally, data analysis of 82,243 participants from 42 countries revealed that men had the highest PPU (Bõthe et al., 2024). Clinical samples showed a comparable frequency and among adult outpatients with OCD lifetime (Fuss et al., 2019) and among adults with Autism Spectrum Disorder (ASD), males had a higher incidence of CSBD than females (Schöttle et al., 2017).

Early treatment studies of CSBD reported higher prevalence of males in treatment for CSBD (Reid et al., 2012a) but not in adolescents in psychiatric treatment (Grant et al., 2007) and Swedish adults (Görts Öberg et al., 2017). The higher rate of males was presumably due to underreporting among women in clinical samples (Derbyshire and Grant, 2015). Despite these differences in prevalence, men and women with CSBD showed similar behavioral patterns and psychological characteristics (Reid et al., 2012b). Table 2 describes epidemiological studies of gender differences in Compulsive Sexual Behavior Disorder (CSBD).

**TABLE 2:** Epidemiological studies of gender differences in compulsive sexual behavior disorder (CSBD).

Author and year	Study design	Sample characteristics (Size/Gender)	Assessment tools	Prevalence rates and sex differences
Walton et al. (2017b)	Cross-sectional	*N* = 510; 52% men, 48% women; mean age of men = 36.52 years (S.D. = 12.66) and women 30.38 years (S.D. = 12.12).	SIS/SES HBI	94 Participants (18.4%) exhibited CSB, including 63 men with a mean age = 32.70 years (S.D. = 13.61) and 31 women with a mean age = 23.06 years (S.D. = 5.85).
Kingston et al. (2018)	Cross-sectional	Students: *N* = 915; 671 females, mean age = 19.5 (S.D. = 2.6) and 244 males, mean age = 20.4 (S.D. = 3.6) Community sample: *N* = 694, 352 females mean age = 25.5 (S.D. = 7) and 342 males, mean age = 28.3 (S.D. = 8.4).	SCS, TSOI, CSBI-C, HBI, and HBCS.	Latent mode factor analysis supported A dimensional latent structure for CSB, particularly in female participants. More males than females met the HBI cutoff in both student and community samples (27.9% vs. 9.2%; 15% vs. 5.7%).
Schöttle et al. (2017)	Cross-sectional	ASD *N* = 96 mean age = 39.2 years (S.D. = 9.5) Healthy control participants, *N* = 96, mean age = 37.9 years (S.D. = 9.7)	HBI QSEB	Men with ASD reported more CSB. No differences between female patients with ASD and female healthy control participants. 17 males with ASD were diagnosed as CSB, only two male healthy control participants scored above the proposed cutoff. No difference was found between female ASD patients and healthy control participants in the rate of CSB.
Müller et al. (2015)	Cross-sectional	*N* = 128; 71.7% men; Men mean age = 26.51 years (S.D. = 6.47). Women mean age = 26.39 years (S.D. = 7.03). 74.2% university students	HBI	3.1% Of participants with CSB. Women exhibited fewer symptoms of CSB than men.
Görts Öberg et al. (2017)	Cross sectional	*N* = 80 16 women, mean age = 29.4 years (S.D. = 9.8), and 64 men, mean age = 40.7 years (S.D. = 9.7) self-identified with CSB	HDSI, SCS, CBOSB	*N* = 40 (50%) of the entire sample 30 men (47%) and 10 women (62%) met the diagnostic criteria for HDSI. Women had a higher total sum score (median = 23) than men (median = 19) and engaged in more frequent risky sexual behavior. Total sum score for women in the HDSI group was higher compared with men with HDSI. Pornography was the most prevalent CSB in men.
Vaillancourt-Morel et al. (2017)	An online convenient sample	*N* = 830 adults 596 women (71.8%), and 234 men (28.2%). Mean age 25.2 years (S.D. = 8) age range 18–78 years old.	CPUI, GMSS, SCS, ASES	Three distinct profiles were identified: Recreational (75.5%): higher sexual satisfaction, lower compulsivity, avoidance, dysfunction; highly distressed Non-compulsive (12.7%): lower sexual satisfaction, low compulsivity, high dysfunction, and avoidance Compulsive (11.8%): lower sexual satisfaction, higher compulsivity, avoidance, and dysfunction. Recreational users were more likely to be women; solitary users were more likely to be highly distressed; men were more likely to be compulsive.
Shimoni et al. (2018)	Cross-sectional	*N* = 267, 186 men, 81 women. Men mean age = 25.23 years (S.D. = 3.75) Women mean age = 32.34 years (S.D. = 3.53) recruited from Internet sites used for finding sexual partners	SAST, BFI	120 Participants, 95 men and 25 women, were classified as CSB and 147 as non-CSB, following criteria defined by Carnes (1991) (SAST score > 6). Men scored higher on CSB than women, were more open to experiences, and were less neurotic. Greater neuroticism was associated with higher scores of SAST in men but not in women.
Dickenson et al. (2018)	A national survey of sexual health and behavior	*N* = 2,325 adults, 1174 (50.5%) women. Mean age = 34 years (S.D. = 9.3).	CSBI-13	8.6% Met the criteria for CSB (score ≥ 35/65), indicating distress/impairment due to difficulty controlling sexual feelings, urges, and behavior Prevalence: 10.3% in men and 7% in women.
Engel et al. (2019)	An online survey	*N* = 1,194 Women- *N* = 564 mean age = 33.83 years (S.D. = 15.25) Men-*N* = 630 mean age = 50.52 years (S.D. = 19.34)	HBI, s-IATsex	360 Individuals, *n* = 74 (13.1%) women; *n* = 286 (45.4%) men, had an HBI-19 sum score of at least 53. A comparison of HBI scores showed higher scores in men mean age = 50.52 (S.D. = 19.34) compared with women mean age = 33.82 (S.D. = 15.25).
Bõthe et al. (2018)	A large-scale psychometric survey study	*N* = 18,034, 6,132 women (34%), mean age = 33.6 years (S.D. = 11.1), age range 18–76 years. Including heterosexual men, LGBTQ men, heterosexual women, LGBTQ women.	HBI	LGBTQ men scored highest on the HBI. LGBTQ men were at higher risk for CSB than women.
Fuss et al. (2019)	Cross-sectional outpatient study	*N* = 539 adult men and women outpatients with current OCD, mean age = 34.8 years (S.D. = 11.8).	CSBD	Eighteen patients (3.3%) reported current CSBD. The rate was higher in male patients compared to female patients. The lifetime prevalence of CSBD in OCD patients was 5.6%, and it was higher in men than in women.
Castro-Calvo et al. (2020)	A community survey study	Sample 1: *N* = 1,581 university students, 43.1% men, 56.9% women. Mean age = 20.58 (S.D. = 2.17)Sample 2: *N* = 1,318 community members, 56.4% men, 43.6% women, Mean age = 34.11 (S.D. = 16.74)	Composite index for CSBD symptoms based on the HBI, SCS, and SAST.	Estimated CSBD prevalence: 10.12% (Sample 1), 7.81% (Sample 2). Individuals with CSBD were mostly heterosexual men, younger, with higher sexual sensation seeking, higher erotophilia, with greater offline and online sexual activity, more depressive and anxious symptoms, and poorer self-esteem compared to those without CSBD.
Levi et al. (2020)	Cross-sectional	Study 1: *N* = 177 143 women, mean age = 32.79 years (S.D. = 9.52) 32 men, mean age = 30.18 years (S.D. = 10.79) Study 2: *N* = 139 98 women, mean age = 24 (S.D. = 5); 41 men, mean age = 25 years (S.D. = 4).	SAST, s-IAT-sex	49 Participants, 11 men and 38 women, were classified with CSB, and 126 as non-CSB, following criteria defined by Carnes (1991) of SAST score > 6. Men had greater scores of CSB than women indicating a significant effect of gender on CSB.
Slavin et al. (2020a)	Population-based study	*N* = 16,823 Hungarian participants above age 18, *N* = 11,191 (66%) males, 5,529 (32%) females, and 103 (0.6%) did not specify a gender. Mean age = 33.26 (S.D. = 0.95) age range between 18 and 76 years.	HBI, SAHQ	Among the sample of 16,823 individuals, 1,202 (7.1%) endorsed CSA (without AASA), including 6% of men and 9.4% of women. 3,399 (20.2%) endorsed AASA (without CSA), including 14.1% of men and 32.4% of women. 1,630 (9.7%) endorsed CSA and AASA, including 4.3% of men and 20.6% of women. Child sexual abuse predicted CSB in both men and women.
Bõthe et al. (2020)	Population-based study	Three non-clinical samples: Study 1: *N* = 14,006, 4,185 women (30%). Mean age 33.2 years (S.D. = 10.9), age range 18–76 years. Study 2: *N* = 483, 252 women (52.2%) Study 3: *N* = 672; 43 women (6.4%). They were recruited from general websites and pornography sites.	FPU, PPU	Three profiles: (1) Non-problematic low-frequency use (68–73%) (2) Non-problematic high-frequency use (19–29%) (3) Problematic high-frequency use (3–8%). Non-problematic and problematic high-frequency-use groups showed differences in CSB, depressive symptoms, boredom susceptibility, self-esteem, uncomfortable feelings regarding pornography, and basic psychological needs. No reported sex differences.
Bõthe et al. (2023)	Population-based study an international survey	*N* = 82,243 participants; mean age = 32.39 years (S.D. = 12.52); most participants were women (*n* = 46,874; 57.0%), followed by men (*n* = 32,549; 39.6%), and gender-diverse individuals (*n* = 2,783; 3.4%). across 42 countries, multiple genders and sexual orientations.	CSBD-19, CSBD-7	4.8% Of the participants were at a high risk of CSBD. Men had the highest scores on the CSBD-19, followed by gender-diverse individuals and women, with a moderate effect size
Bõthe et al. (2024)	Population-based study: An international Survey	*N* = 82,243; mean age = 32.4 years (S.D. = 12.5), a study across 42 countries from five continents	PPCS BPS	Men had the highest PPU scores (mean = 38.55, SD = 19.85), followed by gender-diverse individuals (mean = 32.06, SD = 18.54) and women (mean = 24.24, SD = 11.03), with large effect sizes

Studies are arranged chronologically. AASA, Adolescent/Adult Sexual Abuse; ASES, Arizona Sexual Experiences Scale; ASD, Autism Spectrum Disorder; BFI, Big Five Index; BPS, Brief Pornography Screen; CBOSB, Cognitive and Behavioral Outcomes of Sexual Behavior Scale; CSA, Child Sexual Abuse; CSBD-7, Compulsive Sexual Behavior Disorder Scale-original version (CSBD-19) short version; CSBI-13, Compulsive Sexual Behavior Inventory-13; CPUI, Cyber Pornography Use Inventory; FPU, Frequency of Pornography Use; GMSS, Global Measures of Sexual Satisfaction; HBCS, Hypersexual Behavior Consequences Scale; HBI, Hypersexual Behavior Inventory; HDSI, Hypersexual Disorder Screening Inventory; PPU, Problematic Pornography Use; PPCS, Problematic Pornography Consumption Scale; QSEB, Questionnaire about Sexual Experiences and Behaviors; SAHQ, Sexual Abuse History Questionnaire; SAST, Sexual Addiction Screening Test; SCS, Sexual Compulsivity Scale; SIS/SES, Sexual Inhibition/Sexual Excitation Scales; s-IATsex, Short Internet Addiction Test (sex); TSOI, Total Sexual Outlet Inventory.

## 4 The psychobiology of sexual behavior and compulsive sexual behavior

Little is known regarding the biological basis of individual and sex differences in human sexuality. Fattore et al. (2014) argued that men and women differ in their propensity to addiction involving rewarding stimuli such as sex and food, as well as behaviors like gambling and exercise. Studies on sexual behavior have primarily concentrated on the neurotransmitters, hormones and underlying brain mechanisms that can explain both normative sexual behavior and compulsive sexual behavior. Figure 1 describes a prisma flowchart of study selection and exclusion.

### 4.1 Neurotransmitters and hormones

Sexual activity is regulated by monoamines like dopamine, serotonin and noradrenaline (Chatzittofis et al., 2022). Human sexuality may be associated with genes, in particular genes responsible for dopamine like the D_4_ dopamine receptor, which was associated with novelty seeking, disinhibition, and impulsivity (Congdon et al., 2008), and may influence the initiation of sexual activity (Eisenberg et al., 2007) promiscuity, and infidelity (Garcia et al., 2010). There is further evidence that in males, dopamine is related to human sexuality, and erectile pathways were modulated by dopamine in male clinical patients. Parkinson’s patients who were treated with apomorphine, L-DOPA or bromocriptine reported increased erections, sexual drive and an increased interest in sex (O’Sullivan and Hughes, 1998). Schizophrenic patients who were treated with amantadine which releases dopamine and apomorphine (a dopamine agonist) reported positive effects on erection, sexual desire and satisfaction (Valevski et al., 1998). During human ejaculation, strong activation occurs in the mesencephalic junction, which includes the ventral tegmental area (VTA), a region containing dopaminergic neurons that plays a crucial role in reward processing and motivated behavior (Holstege et al., 2003).

Endocrine diseases, such as hypogonadism, can significantly affect sexual function. Hypogonadism has been associated with hypoactive sexual desire and erectile dysfunction in men (Balercia et al., 2007). In women, alteration of androgen levels can influence sexual desire; excessive androgen production may lead to increased sexual drive and urges. Compulsive sexual behavior in women has been linked to adrenal gland disorders, which can result in loss of life and other serious health complications (Al Awlaqi et al., 2016). Additionally, brain lesions in frontal and temporal lobes, as well as conditions like Kluver-Bucy syndrome, were associated with CSB (Kühn and Gallinat, 2016).

**FIGURE 2:**
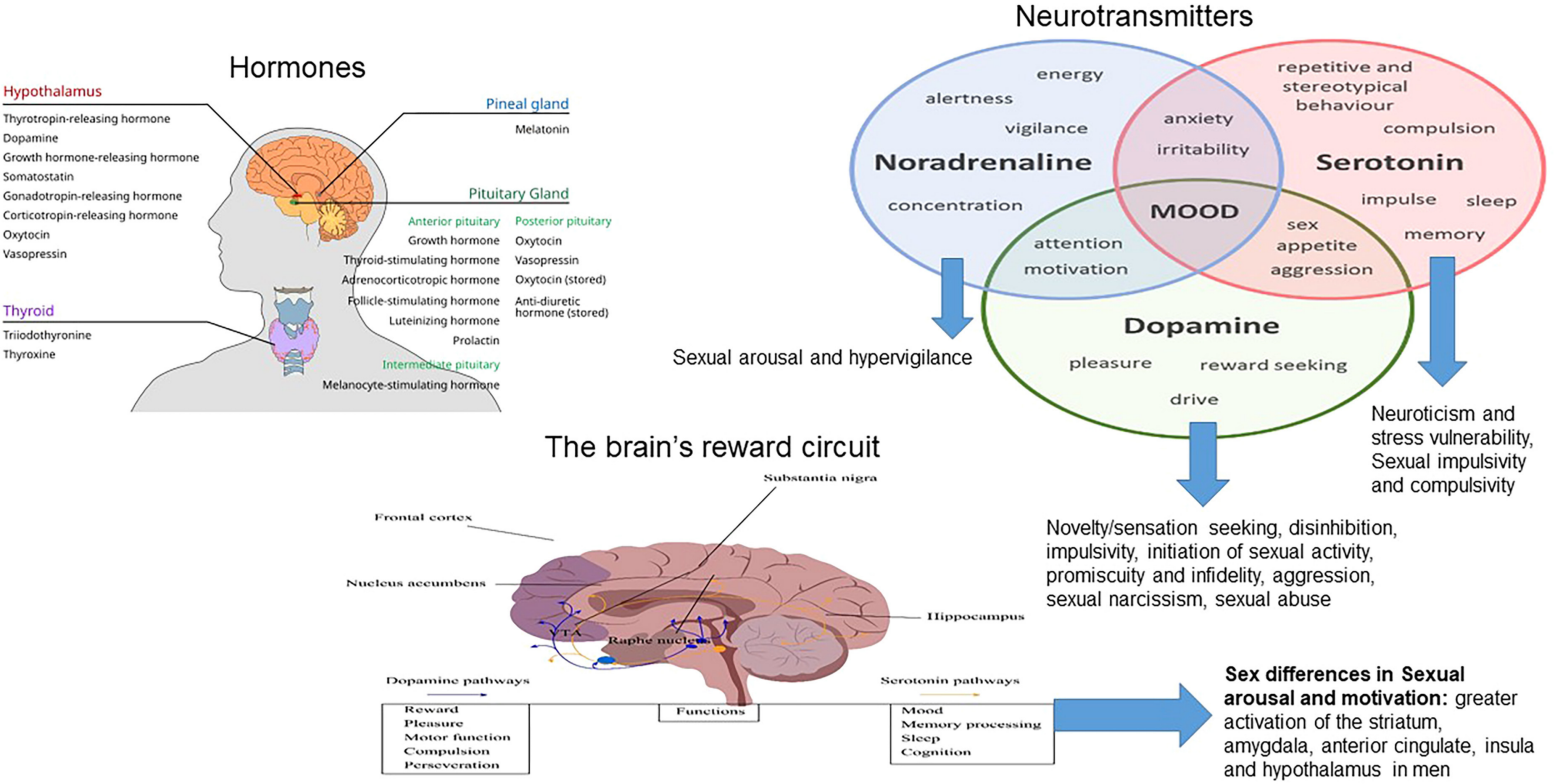
A conceptual framework of the psychobiology of compulsive sexual behavior.

The endocrine systems, particularly the hypothalamus-pituitary adrenal (HPA), the hypothalamus-pituitary–gonadal (HPG) axis, and the oxytocin system were explored recently (Chatzittofis et al., 2022). Studies reported mixed results regarding the hormone cortisol, with some suggesting it inhibits sexual behavior, while others suggest it facilitates sexual behavior (Goldey and van Anders, 2012). The HPG axis, particularly testosterone, plays a key role in sexual activity and has effects on motivation, emotionality, cognition, and autonomic responses (Cunningham et al., 2012; Jordan et al., 2011). However, male patients with CSBD showed no difference in plasma testosterone levels compared with healthy controls (Chatzittofis et al., 2020). The neuropeptide oxytocin, which is involved in prosocial, stress regulation, affiliation behavior, and addiction, was reported as playing a role in sexual behavior (Bancroft, 2005; Heinrichs et al., 2009; Burri et al., 2008; Ross and Young, 2009; Sanna and De Luca, 2021). Oxytocin release increases during penile erection and ejaculation (Argiolas, 1992; Melis et al., 1986; Yanagimoto et al., 1996), and is related to orgasm intensity. Moreover, naloxone induced inhibition of oxytocin shown to reduce sexual satiety, independent of ejaculation (Thackare et al., 2006; Carmichael et al., 1994; Murphy et al., 1990). Figure 2 describes a conceptual framework of the psychobiology of CSBD.

### 4.2 Neuroimaging of human sexuality

Brain imaging studies have examined sexual behavior in both healthy control participants and participants with CSBD and measured an activation in responses to exposure to Visual Sexual Stimuli (VSS) (Kraus et al., 2016b). The brain’s meso-limbic dopamine reward circuit is known to control reward-motivated behavior in humans.

(Schott et al., 2008; Weinstein, 2023). Studies of cue reactivity to substances of abuse showed increased activity in the ventral striatum, and the dorsal anterior cingulate (Kühn and Gallinat, 2011a).

### 4.3 Studies of healthy volunteers

Several studies showed that cue exposure to VSS activated the brain’s reward system, including areas within the limbic and para-limbic system such as the anterior cingulate gyrus and orbitofrontal cortex, striatum, and the amygdala (Sescousse et al., 2013). Studies also showed activation of other areas like the thalamus, hypothalamus, insula, occipital lobe, and parietal lobe in response to VSS. These areas are associated with physical and emotional arousal, visual attention, and motivation (Kühn and Gallinat, 2011b). One of the earliest neuroimaging studies of brain responses to VSS used ^15^O–H_2_O in Positron Emission Tomography (PET) to measure regional cerebral blood flow (rCBF) in 10 young heterosexual males while they watched a pornographic video showing heterosexual intercourse (Bocher et al., 2001). Watching VSS correlated with activation of bilateral infero-posterior extrastriate cortices, the right inferolateral prefrontal cortex, and the midbrain. Similar results were observed by Stoléru et al. (1999). Further studies suggest that the frontal areas of the brain may exert control over reward centers (Georgiadis, 2012; Sescousse et al., 2010). The prefrontal cortex (PFC) sends projections to limbic areas of the brain in order to facilitate a top-down control on execution of goal-directed behaviors (Del Arco and Mora, 2009). The dual system model suggests that risky behavior results from an interaction between the socio-emotional limbic system, and a prefrontal cognitive control system which is important for the understanding of cognitive control over urges (Steinberg, 2010).

The following studies showed specific sex differences in neural responses to sexual arousal and sexual motivation. Male sexual arousal was associated with activation of the visual cortex even when the participants’ eyes were closed while being scanned in PET (Georgiadis et al., 2010). The Insula, amygdala, occipital lobe, sensory-motor area and the hypothalamus were linked to erection in males (Arnow et al., 2002; Ferretti et al., 2005). In contrast, female sexual arousal was associated with increased activity in the dorsal frontal-parietal regions and pre-motor and posterior parietal areas (Georgiadis et al., 2009). During orgasm, brain activity in males and females was similar, involving an activation of the anterior cerebellar vermis and cerebellar nuclei, and deactivation in the ventromedial prefrontal and orbito-frontal cortex, and in the left temporal lobe (Georgiadis et al., 2009). Further evidence showed an activation of the amygdala and hypothalamus during sexual arousal in men, whereas women did not show the same neural activation in response to VSS in fMRI although they rated the movie as sexually arousing Furthermore, men showed an activation of the amygdala during sexual arousal and reduced activation after sexual climax, indicating dynamic changes in reward-related brain regions (Hamann et al., 2004). Men also showed an activation in the ventral tegmental area in the midbrain and the lateral putamen, regions that contain dopaminergic projections that play an important role in the brain’s reward system (Holstege et al., 2003).

Strahler et al. (2018) investigated attentional interference caused by sexually arousing distractors in women and men in fMRI. Participants responding to VSS compared to neutral pictures, and all genders showed longer response times for sexual images. While no sex differences were found in sexual arousal, distractibility or trait sexual motivation. Sexual images activated brain regions associated with motivation and reward. Men showed greater activation of the nucleus caudate, anterior cingulate cortex, and nucleus accumbens compared with women. Sexual motivation correlated with activity in the nucleus caudate, suggesting that VSS activate the reward system. Men’s higher sensitivity to sexual cues and its effects on reward, may therefore increase their vulnerability to compulsive sexual behavior. Finally, there is clinical evidence that indicates that women use sexually explicit material (SEM) less than men; the reasons remain unclear. Stark et al. (2019) investigated neural correlates toward SEM in a phase of expectation phase (sexual or neutral cues) and a phase of presentation phase (sexual or neutral stimuli) in fMRI. SEMs activated the brain’s reward system, and there were sex differences in brain activation in response to sexual cues during the presentation phase, but not during the expectation phase.

### 4.4 Clinical brain imaging studies of CSBD

There are few neuroimaging studies on individuals with compulsive sexual behavior. A study by Voon et al. (2014) examined brain responses to varying sexual content cues in heterosexual males with and without CSB. Using fMRI, CSB participants and control participants were exposed to sexually explicit and non-sexual exciting videos. Individuals with CSB reported greater sexual desire (but not liking) compared to control participants. They also showed an activation of the dorsal anterior cingulate, ventral striatum, and the amygdala during exposure to VSS. Functional connectivity in regions correlated with sexual desire (but not liking) in CSB participants. These regions are part of dopamine reward circuit and cue reactivity, supporting the argument that exposure to sexual cues may be related to vulnerability to compulsive sexual behavior. In their study, sexual desire or “wanting” was dissociated from “liking,” in line with the incentive-salience theory of addiction, suggesting that there is strong wanting but not liking of salient rewards (Robinson and Berridge, 2008). A following study of men with CSB showed increased sexual desire and anterior-cingulate and striatal activation in response to pornographic images compared with those without CSB (Seok and Sohn, 2015). Finally, patients with Parkinson’s-disease with CSB who were exposed to sexual cues showed enhanced sexual desire and enhanced activity in limbic, paralimbic, temporal, occipital, somatosensory, and prefrontal regions implicated in emotional, cognitive, autonomic, visual, and motivational processes compared to those without. Sexual desire correlated with activation of the ventral striatum, anterior cingulate and the orbitofrontal cortices (Politis et al., 2013). Table 3 describes Brain imaging studies of sexual arousal and sex differences.

**TABLE 3:** Brain imaging studies of sexual arousal and sex-differenes.

Study	Participants	Method of stimulation	Activated Brain regions	Sex differences
Bocher et al. (2001)	10 Heterosexual men aged 24 ± 32 (average 27).	Cue exposure to a pornographic video clip, featuring heterosexual intercourse using 15^O^–H_2_O in PET	Bilateral infero-posterior extrastriate cortices, right inferolateral PFC, and the midbrain.	Males only
Arnow et al. (2002)	14 Heterosexual males, age 18 ± 30 years	Cue exposure to explicitly erotic, relaxing, and sports videos, in fMRI.	Activations in the right subinsular region including the claustrum, left caudate and putamen, right middle occipital/ middle temporal gyri, bilateral cingulate gyrus and right sensorimotor and pre-motor regions, and in the right hypothalamus.	Males only
Holstege et al. (2003)	Eleven heterosexual males, mean age, 33; range, 19–45.	Ejaculation compared with sexual stimulation. Manual penile stimulation was performed by the volunteer’s female partner. Imaging using regional cerebral blood flow (rCBF) in PET	Activation in the VTA, the midbrain lateral central tegmental area, zona incerta, subparafascicular nucleus, the ventroposterior, midline, and intralaminar thalamic nuclei, the lateral putamen and adjoining parts of the claustrum.	Males only
Hamann et al. (2004)	Twenty-eight heterosexual participants, 14 women, mean age = 25 years, and 14 men, mean age = 25.9 years.	Participants viewed alternating blocks of four types of stimuli: heterosexual couples engaged in explicit sexual activity (couples stimuli), attractive opposite-sex nudes in modeling poses (opposite-sex stimuli), pleasant social interaction between partially or fully clothed males and females with minimal or no overt sexual content (neutral stimuli; therapeutic massage, dancing, weddings) or a visual fixation cross. In fMRI.	Men and women showed similar activation patterns across multiple brain regions, including ventral striatal regions involved in reward.	Greater activation of the amygdala and hypothalamus during sexual arousal in men, whereas women did not show the same neural activation in response to VSS although they rated the movie as sexually arousing.
Ferretti et al. (2005)	Ten heterosexual men, aged between 21 and 25 years	Viewing erotic scenes in video clips with a long duration, which led to sexual arousal and penile erection, and briefly presented still images, that induced sexual arousal without erection in fMRI.	Activation of the anterior cingulate, insula, amygdala, hypothalamus, and secondary somatosensory cortices which correlated with penile erection. These areas showed dynamic correlation with the time course of sexual response.	Males only
Georgiadis et al. (2009)	11 Males, mean age = 33, range 19–45, and 12 females, mean age = 32, range 21−47.	Cue exposure to VSS using [15*^O^*]−H_2_O in PET	During orgasm, brain activity in males and females was similar, involving an activation of the anterior cerebellar vermis, and cerebellar nuclei, and deactivation in the prefrontal, ventromedial, OFC, and the left temporal lobe.	Male sexual arousal activated the right posterior claustrum, the ventral occipito-temporal region, and the posterior lobe of the cerebellar vermis. Female sexual arousal activated the dorsal frontal-parietal regions and pre-motor and posterior parietal areas. During orgasm, in men, the midbrain and the left lingual gyrus were activated. In women, the right insula was activated.
Georgiadis et al. (2010)	Sixteen heterosexual men, mean age = 29.3 years, range 21–48.6 years with their sexual partners	Two periods of tactile stimulation of the penis and three periods without tactile stimulation during 30 min of scanning. Arterial spin labeling (ASL) measuring rCBF in fMRI in PET.	Stimulation activated the pelvic/genital primary sensorimotor cortex, the claustrum, the anterior part of the middle cingulate cortex (MCC), the dorsal putamen and the Rolandic operculum. Activation of the right insula, basal forebrain, and dorsomedial thalamus, and left posterior cingulate cortex. Decreased rCBF was found in bilateral medial and lateral parts of the PFC, the lateral OFC and the ventromedial PFC, bilateral temporal convexity, and in posterior parts of the parietal cortex. Post-stimulation-activation of the ventral pallidum, extrastriate lingual gyrus, and the occipito-temporal cortex. Decreased rCBF in dorsomedial PFC and vmPFC, and in pregenual and subgenual ACC, antero-ventral hypothalamus, amygdala, and the hippocampus.	Males only
Sescousse et al. (2010)	Eighteen right-handed heterosexual males, age = 24 ± 3.3 years.	A reward anticipation task with erotic or monetary reward in fMRI.	Erotic stimuli activated the Medial OFC, including the medial orbital gyrus, the straight gyrus, and the most ventral part of the superior frontal gyrus, and the bilateral amygdala.	Males only
Politis et al. (2013)	Twenty-four non-demented patients with idiopathic Parkinson’s disease. 11 males and 1 female. Mean age = 55.2 (S.D. = 9.2). Twelve diagnosed with CSB and 12 control without history of CSB 10 males and 2 females. Mean age = 62.3 (S.D. = 9.7).	Five types of color images were presented in a block design: (1) dopaminergic drugs cues; (2) appetizing food cues; (3) money and gambling cues; (4) sexual cues; and (5) neutral cues in fMRI.	Patients with Parkinson’s-disease with CSB who were exposed to sexual cues showed enhanced sexual desire and activation in limbic, paralimbic, temporal, occipital, somatosensory, and prefrontal regions compared to those without. Sexual desire correlated with activation of the ventral striatum, ACC and the OFC. When the patients with Parkinson’s disease with CSB were OFF medication, inactivation during presentation of sexual cues relative to rest.	Too few females to enable comparison.
Voon et al. (2014)	Nineteen heterosexual men with CSB, mean age = 25.61 years (S.D. = 4.77) and 19 age-matched controls Mean age = 23.17 years (S.D. = 5.38) heterosexual male healthy volunteers without CSB	Cue exposure to subjects viewed video clips presented in a counter- balanced order of 5 conditions: explicit sexual (VSS), erotic, non-sexual exciting, money and neutral videos in fMRI	Men with CSB reported greater sexual desire (but not liking) compared to control participants. They also showed an activation of the dorsal ACC, ventral striatum, and the amygdala during exposure to VSS. Functional connectivity in regions correlated with sexual desire (but not liking) in CSB participants.	Males only.
Seok and Sohn (2015)	23 Heterosexual males participants in the problematic CSB group, mean age = 26.12 years (S.D. = 4.11) and 22 heterosexual males in the control group, mean age = 26.27 years (S.D. = 3.39).	Cue exposure to pornographic photos, affective photos and neutral photos in fMRI.	The CSB group showed activation in the right dorsal ACC, bilateral thalami, left caudate nucleus, right DLPFC, and right supra-marginal gyrus relative to the activation in the control group during exposure to sexual stimuli compared with non-sexual stimuli.	Males only.
Strahler et al. (2018)	97 Heterosexual participants, 50 women mean age = 25.1 (S.D. = 4.88) and 47 men, mean age = 25.43 (S.D. = 4.15) age range (20–45) mainly students.	Cue exposure- indicating the orientation of two lines (equal or unequal) next to an explicit sexual as compared to a neutral) picture in fMRI.	Participants responding to VSS compared to neutral pictures, showed longer response times for sexual images. While no sex differences were found in sexual arousal, distractibility or trait sexual motivation. Sexual images activated brain regions associated with motivation and reward.	Men showed greater activation of the nucleus caudate, anterior cingulate cortex, and nucleus accumbens compared with women. Sexual motivation correlated with activity in the nucleus caudate, suggesting that VSS activate the reward system.
Stark et al. (2019)	70 Participants, Heterosexual and bisexual, 33 females Mean age = 24.24 years (S.D. = 4.47), and 37 males, mean age = 27.05 years (S.D. = 4.40), age range (19–44).	Neural correlates toward VSS in the expectation phase (sexual or neutral cues) and a presentation phase (sexual or neutral stimuli) in fMRI	VSS activated the ACC, Caudate, Putamen, Pallidum, OFC, amygdala, thalamus, hypothalamus, insula, and VTA, areas of the brain’s reward system. Cues, which predict VSS, activated the same brain regions as VSS.	Sex differences in brain activation in response to sexual cues during the presentation phase, but not during the expectation phase. Men showed higher activations than women for the contrast sexual -neutral cues in the vental ACC, caudate, pallidum, OFC, and insula. Women showed greater activation than men in the thalamus and hypothalamus.

Studies are arranged chronologically. ACC, Anterior Cingulate Cortex; ASL, Arterial Spin Labeling; DLPFC, Dorso Lateral Prefrontal Cortex; fMRI, Functional Magnetic Resonance Imaging; OFC, Orbito Frontal Cortex; PFC, Prefrontal Cortex; PET, Positron Emission Tomography; rCBF, Regional Cerebral Blood Flow; VTA, Ventral tegmental area; VSS, Visual Sexual Stimuli.

## 5 Personality trait level vulnerabilities, impulsivity, and neuroticism

Studies on CSBD among men and women who seek sexual partners or participate in self-help groups indicate that impulsivity and psychiatric comorbidity are common across both sexes. A meta-analysis examining impulsivity, PPU and sex differences revealed that PPU positively correlated with impulsivity and sensation seeking (Bocci Benucci et al. (2024). Analysis of data from Wave-2 of the National Epidemiologic Survey on Alcohol and Related Conditions (NESARC), including 34,653 U.S. participants showed a high prevalence of sexual impulsivity (14.7%), with men showing higher percentage than women (19% vs. to 11%) (Erez et al., 2014). In women, sexual impulsivity was particularly correlated with social phobia, alcohol abuse or dependence, and a range of personality disorders. Factors such as neuroticism and stress vulnerability play a significant role in CSBD symptoms in women. A study of a large sample of individuals who used internet sites to find sexual partners, showed that men scored higher than women on measures of sex addiction and openness to experiences, but lower on neuroticism (Shimoni et al., 2018). Furthermore, neuroticism positively correlated with sex addiction in men but not in women (Shimoni et al., 2018).

## 6 Related constructs

### 6.1 Sexual abuse and sexual narcissism

Previous small-scale cross-sectional studies showed high rates of childhood trauma and abuse particularly in women seeking treatment for CSB (Opitz et al., 2009; Perera et al., 2009; Ross, 1996; Schwartz and Southern, 2000; Tedesco and Bola, 1997; Weiss, 2000, see Slavin et al., 2020b for a review). Women who used social networking sites for finding sexual partners reported that childhood abuse and neglect were associated with CSB (Ashkenazi et al., 2022). Finally, two samples of U.S. university young adults (70% women), confirmed a relationship between CSA and CSB with distress more central for men, whereas trauma was more central for women. Dissatisfaction was strongly connected to relapse in men and to negative consequences in women (Scoglio et al., 2025).

Men scored higher on measures of sexual exploitation and risks for engaging in sexually coercive perpetration than women (Basting et al., 2023). Childhood trauma positively correlated with sexual narcissism and CSB among participants in sex help groups, and sexual narcissism increased the likelihood of CSB, and served as a mediating factor between trauma and compulsive sexual behavior (Yaakov and Weinstein, 2025), a finding with important implications for treatment.

## 7 Discussion

The studies reviewed so far show evidence for sex differences in human sexual arousal and in compulsive sexual behavior, but this evidence should be taken with caution. Sexual activity is regulated by monoamines like dopamine, serotonin, and noradrenaline and in particular with dopamine, which is associated with novelty seeking, disinhibition, impulsivity, the initiation of sexual activity, promiscuity and infidelity. In males, dopamine is related to sexuality, and erection in clinical patients. Hormones like androgens play a role in human sexuality, and can influence sexual desire, sexual drive, and urges. Furthermore, endocrine systems, like the HPA, HPG axis, and oxytocin were implicated in sexual behavior although there were mixed results regarding levels of cortisol, testosterone and oxytocin in CSBD (Chatzittofis et al., 2022).

Neuroimaging studies outlined sex differences in neural responses to sexual stimuli in fMRI. Men’s higher sensitivity to the rewarding value of sexual cues may contribute to their increased risk of addictive or compulsive sexual behavior. Several studies have shown sex differences in sexual arousal and sexual motivation. There is also evidence for an activation of reward areas in the brain in response to sexually explicit stimuli in CSBD. Studies on compulsive sexual behavior consistently report a higher prevalence of CSBD among men. However, these are mainly cross-sectional studies involving convenience samples, and the difference may be influenced by cultural factors rather than biological factors. Pornography and cybersex are frequent sexual activities particularly among males with CSBD (Weinstein et al., 2015; Borgogna et al., 2022; Villena-Moya et al., 2025). Neuroticism and stress vulnerability also play a significant role in CSBD symptoms in women, and childhood trauma and abuse, is prominent among women seeking treatment for CSBD.

This review provides new insights into the neurobiological basis of sex differences in sexual arousal, sexual behavior and compulsive sexual behavior. There are few and contradictory findings of sex differences in hormones and neurotransmitters. The few brain imaging studies indicate sex differences in brain response to visual sexual stimuli and activation of the brain’s reward circuit in individuals with CSB. There are currently no brain imaging studies on sex differences in this population. Further research is required on sex differences in the brain’s activity, hormones and neurotransmitters in men and women with compulsive sexual behavior.

## 8 Limitations

The studies reported so far were mainly cross-sectional, and therefore, no causal inferences can be made. The majority of epidemiological studies are small-scale, convenience samples, and there are very few large, population-based studies on CSBD. Most brain imaging studies investigated exposure to visual sexual stimuli in healthy male volunteers. The activation studies in response to sexually explicit stimuli in CSBD were done in males and not in female participants. The findings on sex differences are therefore based mainly on heterogeneous healthy male volunteers and should not be overextended to CSBD.

## 9 Conclusion

CSB affects men more frequently than women, a difference that may be influenced by cultural factors. Human sexuality is associated with neurotransmitters and hormones, in particular dopaminergic genes related to reward. Brain imaging studies showed higher reactivity to sexual cues and their effects on reward in men, which may increase their vulnerability to compulsive sexual behavior. There is little and contradictory evidence about sex differences in treatment for CSBD. Further research is required on sex differences in brain activity, hormones and neurotransmitters in men and women with CSB.
